# [3-({(*E*)-2-[(4-Fluorophenyl)carbamo­thioyl]hydrazinylidene}methyl)-4-hy­droxy­benzyl]methyl­triphenyl­phos­phonium chloride

**DOI:** 10.1107/S1600536811047945

**Published:** 2011-11-16

**Authors:** Saravana Kumar Sinniah, Kong Wai Tan, Kae Shin Sim, Seik Weng Ng, Edward R. T. Tiekink

**Affiliations:** a Institute of Biological Sciences, University of Malaya, 50603 Kuala Lumpur, Malaysia; b Department of Chemistry, University of Malaya, 50603 Kuala Lumpur, Malaysia; c Chemistry Department, Faculty of Science, King Abdulaziz University, PO Box 80203 Jeddah, Saudi Arabia

## Abstract

The cation in the title salt, C_33_H_28_FN_3_OPS^+^·Cl^−^, is highly twisted with the phospho­nium group occupying a position almost normal to the central hydroxyl­benzene ring [P—C—C—C tosrsion angle = −100.9 (3)°], and with the hydrazone substituent twisted out of the plane [C—C—C—N torsion angle = 13.1 (4)°]. The fluoro­benzene ring is twisted out of the plane of the adjacent thio­urea residue, forming a dihedral angle of 51.69 (10)°. The configuration about the C=N bond [1.281 (4) Å] is *E*, the O—H and N—H hydrogen atoms are *syn*, and in the thio­urea residue, the N—H hydrogen atoms are *anti*, allowing for the formation of an intra­molecular N—H⋯N hydrogen bond. In the crystal, dimeric aggregates mediated by N—H⋯S bonds are formed, which are linked to the Cl^−^ anions by O—H⋯Cl hydrogen bonds. The four-component aggregates are linked into a three-dimensional structure by C—H⋯Cl inter­actions.

## Related literature

For the crystal structure of the related compound salicyl­aldehyde 4-phenyl­thio­semicarbazone, see: Rubčić *et al.* (2008 ▶). For the anti-tumour, anti-viral and anti-fungal activity of thio­semicarbazones, see: Kalinowski *et al.* (2009 ▶); Beraldo & Gambino (2004 ▶). For the biological properties of triphenyl­phospho­nium-containing Schiff bases, see: Shahabadi *et al.* (2010 ▶).
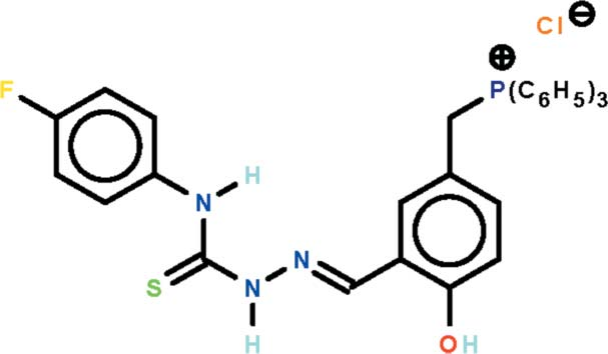


## Experimental

### 

#### Crystal data

C_33_H_28_FN_3_OPS^+^·Cl^−^
*M* *_r_* = 600.06
Monoclinic, 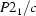

*a* = 17.5495 (6) Å
*b* = 9.4617 (3) Å
*c* = 19.0569 (6) Å
β = 107.298 (4)°
*V* = 3021.24 (17) Å^3^
*Z* = 4
Mo *K*α radiation
μ = 0.29 mm^−1^
*T* = 100 K
0.30 × 0.25 × 0.20 mm

#### Data collection

Agilent SuperNova Dual diffractometer with Atlas detector
Absorption correction: multi-scan (*CrysAlis PRO*; Agilent, 2010 ▶) *T*_min_ = 0.919, *T*_max_ = 0.945
12024 measured reflections
6178 independent reflections
4374 reflections with *I* > 2σ(*I*)
*R*_int_ = 0.040

#### Refinement

*R*[*F* ^2^ > 2σ(*F* ^2^)] = 0.054
*wR*(*F* ^2^) = 0.154
*S* = 1.04
6178 reflections
382 parameters
3 restraints
H atoms treated by a mixture of independent and constrained refinement
Δρ_max_ = 0.62 e Å^−3^
Δρ_min_ = −0.38 e Å^−3^

### 

Data collection: *CrysAlis PRO* (Agilent, 2010 ▶); cell refinement: *CrysAlis PRO*; data reduction: *CrysAlis PRO*; program(s) used to solve structure: *SHELXS97* (Sheldrick, 2008 ▶); program(s) used to refine structure: *SHELXL97* (Sheldrick, 2008 ▶); molecular graphics: *X-SEED* (Barbour, 2001 ▶) and *DIAMOND* (Brandenburg, 2006 ▶); software used to prepare material for publication: *publCIF* (Westrip, 2010 ▶).

## Supplementary Material

Crystal structure: contains datablock(s) general, I. DOI: 10.1107/S1600536811047945/hg5137sup1.cif

Structure factors: contains datablock(s) I. DOI: 10.1107/S1600536811047945/hg5137Isup2.hkl

Additional supplementary materials:  crystallographic information; 3D view; checkCIF report

## Figures and Tables

**Table 1:** Hydrogen-bond geometry (Å, °)

*D*—H⋯*A*	*D*—H	H⋯*A*	*D*⋯*A*	*D*—H⋯*A*
N3—H3⋯N1	0.87 (1)	2.16 (3)	2.580 (4)	109 (3)
O1—H1⋯Cl1	0.84 (1)	2.17 (1)	3.005 (2)	173 (4)
N2—H2⋯S1^i^	0.88 (1)	2.58 (2)	3.429 (3)	162 (3)
C6—H6⋯Cl1^ii^	0.95	2.69	3.572 (3)	154
C19—H19a⋯Cl1^ii^	0.99	2.51	3.488 (3)	168
C19—H19b⋯Cl1^iii^	0.99	2.59	3.553 (3)	165

Symmetry codes: (i) 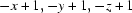; (ii) 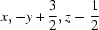; (iii) 
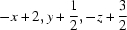
.

**Fig. 1.**
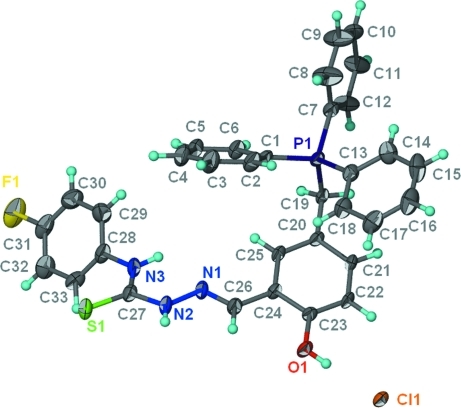
The molecular structures of the ions comprising the asymmetric unit of (I) showing the atom-labelling scheme and displacement ellipsoids at the 70% probability level.

**Fig. 2.**
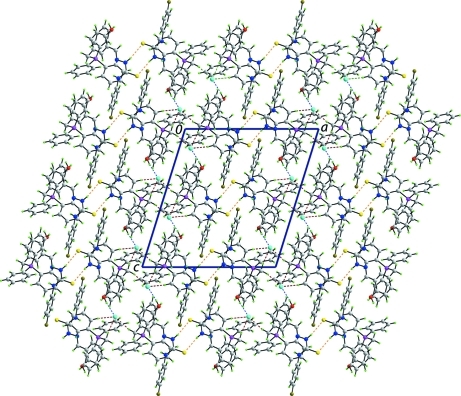
A view in projection down the b axis of the crystal packing in (I) highlighting the mode of association between the constituent ions. The N—H···S, O—H···Cl and C—H···Cl interactions are shown as orange, blue and brown dashed lines, respectively.

**Fig. 3.**
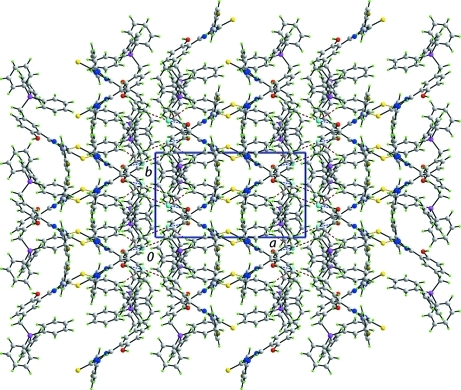
A view in projection down the c axis of the crystal packing in (I) highlighting the stacking of layers along the a-direction. The N—H···S, O—H···Cl and C—H···Cl interactions are shown as orange, blue and brown dashed lines, respectively.

## supplementary crystallographic information

### Comment

As part of efforts in improving the water solubility and biological properties of thiosemicarbazones (Kalinowski *et al.*, 2009; Beraldo & Gambino, 2004), we report herein a new thiosemicarbazone molecule characterized as its Cl^-^ salt, (I), containing a cationic triphenylphosphonium moiety, which is known to exhibit biological properties (Shahabadi *et al.*, 2010). A related structure has been reported previously (Rubčić *et al.*, 2008).

The components of the salt, (I), are illustrated in Fig. 1. With respect to the central hydroxybenzene ring in the cation, the phosphonium-P atom lies in a position almost perpendicular to the ring with the P1—C19—C20—C21 being -100.9 (3)°. On the other side, the hydrazone residue is twisted out of the central plane, with the C25—C24—C26—N1 torsion angle = 13.1 (4)°. The terminal fluorobenzene ring is significantly twisted out of the plane through the adjacent thiourea residue forming a dihedral angle of 51.69 (10)°. The configuration about the C26═N1 bond [1.281 (4) Å] is *E*. While the O—H and N—H hydrogen atoms are *syn*, in the thiourea residue, the N—H hydrogen atoms are *anti*. The latter allows for the formation of an intramolecular N—H···N hydrogen bond, Table 1.

The crystal packing features centrosymmetric {···HNCS}_2_ synthons, Table 1. Two Cl^-^ anions are linked to the resulting dimeric aggregates *via* O—H···Cl hydrogen bonds, with the neutral four component aggregates linked into the three-dimensional architecture by C—H···Cl interactions, Fig. 2 and Table 1. Globally, the crystal structure comprises rows of hydrogen bonded thiourea residues sandwiched by the hydrazone and phosphonium substituents, with the sandwiches stacking along the *a* axis, Fig. 3.

### Experimental

(3-Formyl-4-hydroxy-phenyl)methyl-triphenyl-phosphonium chloride (0.382 g, 1 mmol) was dissolved in ethanol (30 ml) and added to an ethanolic solution (20 ml) of 4-fluorophenyl-3-thiosemicarbazide (0.18 5 g, 1 mmol). The reaction mixture was refluxed for 4 h and the title compound separated as a yellow powder upon cooling. Recrystallization from ethanol afforded yellow crystals.

### Refinement

Carbon-bound H-atoms were placed in calculated positions [C—H 0.95 to 0.99 Å, *U*_iso_(H) = 1.2*U*_eq_(C)] and were included in the refinement in the riding model approximation. The O—H and N—H H-atoms were located in a difference map and refined with distance restraints of 0.84±0.01 and 0.88±0.01 Å, respectively, and with unrestrained *U*_iso_(H).

### Crystal data

**Table:**

C_33_H_28_FN_3_OPS^+^·Cl^−^	*F*(000) = 1248
*M**_r_* = 600.06	*D*_x_ = 1.319 Mg m^−^^3^
Monoclinic, *P*2_1_/*c*	Mo *K*α radiation, λ = 0.71073 Å
Hall symbol: -P 2ybc	Cell parameters from 3679 reflections
*a* = 17.5495 (6) Å	θ = 2.4–29.2°
*b* = 9.4617 (3) Å	µ = 0.29 mm^−^^1^
*c* = 19.0569 (6) Å	*T* = 100 K
β = 107.298 (4)°	Prism, yellow
*V* = 3021.24 (17) Å^3^	0.30 × 0.25 × 0.20 mm
*Z* = 4	

### Data collection

**Table:**

Agilent SuperNova Dual diffractometer with Atlas detector	6178 independent reflections
Radiation source: SuperNova (Mo) X-ray Source	4374 reflections with *I* > 2σ(*I*)
Mirror	*R*_int_ = 0.040
Detector resolution: 10.4041 pixels mm^-1^	θ_max_ = 26.5°, θ_min_ = 2.4°
ω scan	*h* = −17→22
Absorption correction: multi-scan (*CrysAlis PRO*; Agilent, 2010)	*k* = −11→9
*T*_min_ = 0.919, *T*_max_ = 0.945	*l* = −23→16
12024 measured reflections	

### Refinement

**Table:**

Refinement on *F*^2^	Primary atom site location: structure-invariant direct methods
Least-squares matrix: full	Secondary atom site location: difference Fourier map
*R*[*F*^2^ > 2σ(*F*^2^)] = 0.054	Hydrogen site location: inferred from neighbouring sites
*wR*(*F*^2^) = 0.154	H atoms treated by a mixture of independent and constrained refinement
*S* = 1.04	*w* = 1/[σ^2^(*F*_o_^2^) + (0.0639*P*)^2^ + 2.4147*P*] where *P* = (*F*_o_^2^ + 2*F*_c_^2^)/3
6178 reflections	(Δ/σ)_max_ = 0.001
382 parameters	Δρ_max_ = 0.62 e Å^−^^3^
3 restraints	Δρ_min_ = −0.38 e Å^−^^3^

### Special details

**Table:**

Geometry. All s.u.'s (except the s.u. in the dihedral angle between two l.s. planes) are estimated using the full covariance matrix. The cell s.u.'s are taken into account individually in the estimation of s.u.'s in distances, angles and torsion angles; correlations between s.u.'s in cell parameters are only used when they are defined by crystal symmetry. An approximate (isotropic) treatment of cell s.u.'s is used for estimating s.u.'s involving l.s. planes.
Refinement. Refinement of *F*^2^ against ALL reflections. The weighted *R*-factor *wR* and goodness of fit *S* are based on *F*^2^, conventional *R*-factors *R* are based on *F*, with *F* set to zero for negative *F*^2^. The threshold expression of *F*^2^ > 2σ(*F*^2^) is used only for calculating *R*-factors(gt) *etc*. and is not relevant to the choice of reflections for refinement. *R*-factors based on *F*^2^ are statistically about twice as large as those based on *F*, and *R*- factors based on ALL data will be even larger.

### Fractional atomic coordinates and isotropic or equivalent isotropic displacement parameters (Å^2^)

**Table:**

	*x*	*y*	*z*	*U*_iso_*/*U*_eq_	
Cl1	0.91349 (4)	0.62474 (8)	0.85886 (4)	0.02368 (19)	
P1	0.83503 (5)	1.15927 (8)	0.50731 (4)	0.0200 (2)	
S1	0.48397 (5)	0.43111 (9)	0.38203 (4)	0.0283 (2)	
F1	0.58227 (13)	0.3645 (3)	0.08217 (11)	0.0512 (6)	
O1	0.78340 (13)	0.6681 (2)	0.71688 (12)	0.0280 (5)	
N1	0.67395 (15)	0.6310 (3)	0.49955 (14)	0.0253 (6)	
N2	0.60362 (15)	0.5553 (3)	0.47947 (14)	0.0262 (6)	
N3	0.61878 (16)	0.5438 (3)	0.36568 (14)	0.0276 (6)	
C1	0.74010 (17)	1.1072 (3)	0.44749 (15)	0.0212 (6)	
C2	0.66882 (19)	1.1318 (4)	0.46326 (18)	0.0316 (8)	
H2A	0.6687	1.1812	0.5066	0.038*	
C3	0.5978 (2)	1.0833 (4)	0.41479 (19)	0.0386 (9)	
H3A	0.5487	1.0983	0.4253	0.046*	
C4	0.5985 (2)	1.0125 (4)	0.35068 (19)	0.0368 (8)	
H4	0.5501	0.9769	0.3185	0.044*	
C5	0.66894 (19)	0.9939 (3)	0.33374 (18)	0.0301 (7)	
H5	0.6688	0.9491	0.2891	0.036*	
C6	0.73978 (19)	1.0410 (3)	0.38218 (17)	0.0266 (7)	
H6	0.7885	1.0280	0.3708	0.032*	
C7	0.87455 (18)	1.2911 (3)	0.46083 (16)	0.0245 (7)	
C8	0.8314 (2)	1.4157 (4)	0.4393 (3)	0.0535 (12)	
H8	0.7814	1.4273	0.4482	0.064*	
C9	0.8606 (2)	1.5209 (4)	0.4055 (3)	0.0553 (12)	
H9	0.8313	1.6061	0.3919	0.066*	
C10	0.9327 (2)	1.5034 (4)	0.39112 (18)	0.0351 (8)	
H10	0.9522	1.5757	0.3664	0.042*	
C11	0.9762 (2)	1.3819 (4)	0.4124 (2)	0.0373 (9)	
H11	1.0258	1.3699	0.4025	0.045*	
C12	0.9474 (2)	1.2765 (3)	0.4485 (2)	0.0352 (8)	
H12	0.9782	1.1937	0.4649	0.042*	
C13	0.82929 (18)	1.2290 (3)	0.59315 (17)	0.0247 (7)	
C14	0.8756 (2)	1.3467 (4)	0.62295 (19)	0.0340 (8)	
H14	0.9023	1.3982	0.5945	0.041*	
C15	0.8824 (2)	1.3879 (4)	0.6947 (2)	0.0420 (9)	
H15	0.9143	1.4672	0.7153	0.050*	
C16	0.8437 (2)	1.3158 (4)	0.7353 (2)	0.0427 (9)	
H16	0.8484	1.3460	0.7839	0.051*	
C17	0.7978 (2)	1.1993 (4)	0.70702 (19)	0.0372 (9)	
H17	0.7711	1.1495	0.7362	0.045*	
C18	0.7904 (2)	1.1543 (4)	0.63557 (18)	0.0311 (8)	
H18	0.7591	1.0736	0.6159	0.037*	
C19	0.89916 (17)	1.0055 (3)	0.52567 (15)	0.0187 (6)	
H19A	0.8972	0.9582	0.4788	0.022*	
H19B	0.9549	1.0350	0.5499	0.022*	
C20	0.87243 (17)	0.9036 (3)	0.57481 (15)	0.0202 (6)	
C21	0.91367 (18)	0.8996 (3)	0.64994 (16)	0.0229 (7)	
H21	0.9612	0.9532	0.6684	0.027*	
C22	0.88599 (18)	0.8185 (3)	0.69762 (16)	0.0241 (7)	
H22	0.9151	0.8154	0.7483	0.029*	
C23	0.81585 (18)	0.7415 (3)	0.67172 (16)	0.0213 (6)	
C24	0.77585 (17)	0.7385 (3)	0.59589 (16)	0.0207 (6)	
C25	0.80530 (17)	0.8198 (3)	0.54838 (16)	0.0205 (6)	
H25	0.7788	0.8175	0.4971	0.025*	
C26	0.70392 (18)	0.6542 (3)	0.56854 (17)	0.0242 (7)	
H26	0.6788	0.6159	0.6021	0.029*	
C27	0.57265 (18)	0.5127 (3)	0.40903 (16)	0.0244 (7)	
C28	0.60814 (18)	0.4968 (4)	0.29226 (16)	0.0272 (7)	
C29	0.6181 (2)	0.5918 (4)	0.24096 (19)	0.0366 (8)	
H29	0.6300	0.6879	0.2542	0.044*	
C30	0.6108 (2)	0.5470 (4)	0.1695 (2)	0.0392 (9)	
H30	0.6181	0.6110	0.1337	0.047*	
C31	0.59293 (19)	0.4090 (4)	0.15287 (18)	0.0344 (8)	
C32	0.5849 (2)	0.3102 (4)	0.20287 (19)	0.0355 (8)	
H32	0.5738	0.2140	0.1894	0.043*	
C33	0.59363 (19)	0.3568 (3)	0.27397 (17)	0.0292 (7)	
H33	0.5895	0.2911	0.3104	0.035*	
H1	0.8166 (17)	0.658 (4)	0.7584 (10)	0.044 (11)*	
H2	0.576 (2)	0.541 (4)	0.5103 (18)	0.055 (12)*	
H3	0.6576 (14)	0.604 (3)	0.3836 (17)	0.033 (10)*	

### Atomic displacement parameters (Å^2^)

**Table:**

	*U*^11^	*U*^22^	*U*^33^	*U*^12^	*U*^13^	*U*^23^
Cl1	0.0248 (4)	0.0288 (4)	0.0148 (3)	−0.0007 (3)	0.0017 (3)	0.0035 (3)
P1	0.0206 (4)	0.0245 (4)	0.0136 (4)	−0.0001 (3)	0.0030 (3)	0.0014 (3)
S1	0.0221 (4)	0.0395 (5)	0.0211 (4)	−0.0075 (4)	0.0031 (3)	−0.0067 (3)
F1	0.0479 (13)	0.0864 (17)	0.0230 (11)	−0.0020 (12)	0.0162 (10)	−0.0122 (11)
O1	0.0261 (12)	0.0405 (13)	0.0144 (11)	−0.0072 (10)	0.0014 (10)	0.0073 (10)
N1	0.0207 (13)	0.0324 (15)	0.0190 (13)	−0.0064 (12)	0.0002 (11)	−0.0023 (11)
N2	0.0196 (13)	0.0397 (16)	0.0166 (14)	−0.0095 (12)	0.0011 (11)	−0.0033 (11)
N3	0.0259 (15)	0.0371 (16)	0.0173 (14)	−0.0111 (13)	0.0024 (12)	−0.0043 (12)
C1	0.0221 (15)	0.0267 (16)	0.0127 (14)	0.0000 (13)	0.0021 (12)	0.0062 (12)
C2	0.0315 (18)	0.044 (2)	0.0186 (16)	−0.0004 (16)	0.0064 (14)	0.0002 (14)
C3	0.0254 (18)	0.060 (2)	0.0289 (19)	−0.0069 (17)	0.0061 (15)	−0.0011 (17)
C4	0.0273 (18)	0.054 (2)	0.0244 (18)	−0.0106 (17)	0.0003 (15)	0.0022 (16)
C5	0.0314 (18)	0.0347 (18)	0.0204 (16)	0.0019 (15)	0.0017 (14)	−0.0030 (14)
C6	0.0241 (16)	0.0345 (18)	0.0205 (16)	−0.0005 (14)	0.0055 (14)	−0.0009 (13)
C7	0.0245 (16)	0.0286 (17)	0.0188 (16)	−0.0016 (14)	0.0037 (13)	0.0038 (13)
C8	0.032 (2)	0.053 (2)	0.079 (3)	0.0109 (19)	0.022 (2)	0.032 (2)
C9	0.039 (2)	0.049 (2)	0.078 (3)	0.013 (2)	0.018 (2)	0.037 (2)
C10	0.040 (2)	0.037 (2)	0.0269 (18)	−0.0071 (17)	0.0080 (16)	0.0106 (15)
C11	0.047 (2)	0.0324 (19)	0.043 (2)	0.0008 (17)	0.0297 (19)	0.0060 (16)
C12	0.045 (2)	0.0244 (17)	0.043 (2)	0.0091 (16)	0.0237 (18)	0.0107 (15)
C13	0.0281 (17)	0.0258 (16)	0.0199 (16)	0.0023 (14)	0.0067 (14)	−0.0028 (13)
C14	0.0306 (18)	0.0364 (19)	0.036 (2)	−0.0020 (16)	0.0113 (16)	−0.0081 (15)
C15	0.0318 (19)	0.049 (2)	0.042 (2)	0.0003 (18)	0.0047 (17)	−0.0235 (18)
C16	0.044 (2)	0.052 (2)	0.029 (2)	0.0047 (19)	0.0053 (18)	−0.0166 (17)
C17	0.035 (2)	0.052 (2)	0.0255 (19)	0.0059 (18)	0.0097 (16)	−0.0017 (16)
C18	0.0321 (18)	0.0364 (19)	0.0235 (17)	0.0009 (15)	0.0063 (15)	−0.0031 (14)
C19	0.0172 (14)	0.0256 (15)	0.0120 (14)	−0.0004 (12)	0.0023 (12)	−0.0010 (12)
C20	0.0233 (15)	0.0217 (15)	0.0139 (14)	0.0032 (13)	0.0030 (12)	0.0010 (12)
C21	0.0200 (15)	0.0272 (16)	0.0160 (15)	−0.0027 (13)	−0.0033 (12)	−0.0003 (12)
C22	0.0264 (16)	0.0315 (17)	0.0103 (14)	−0.0028 (14)	−0.0010 (13)	0.0006 (12)
C23	0.0216 (15)	0.0254 (16)	0.0163 (15)	−0.0026 (13)	0.0048 (12)	0.0032 (12)
C24	0.0198 (15)	0.0254 (16)	0.0152 (14)	0.0010 (13)	0.0025 (12)	0.0010 (12)
C25	0.0220 (15)	0.0251 (16)	0.0121 (14)	0.0015 (13)	0.0015 (12)	−0.0019 (12)
C26	0.0222 (16)	0.0312 (17)	0.0184 (16)	−0.0036 (13)	0.0046 (13)	−0.0001 (13)
C27	0.0243 (16)	0.0271 (17)	0.0186 (15)	0.0005 (14)	0.0016 (13)	−0.0009 (13)
C28	0.0229 (16)	0.0398 (19)	0.0176 (16)	−0.0025 (15)	0.0037 (13)	−0.0017 (14)
C29	0.041 (2)	0.039 (2)	0.0296 (19)	−0.0111 (17)	0.0116 (16)	−0.0011 (15)
C30	0.041 (2)	0.051 (2)	0.0283 (19)	−0.0029 (18)	0.0139 (17)	0.0076 (17)
C31	0.0265 (17)	0.062 (2)	0.0174 (16)	0.0049 (17)	0.0099 (14)	−0.0044 (16)
C32	0.036 (2)	0.044 (2)	0.0276 (19)	0.0017 (17)	0.0125 (16)	−0.0088 (16)
C33	0.0315 (18)	0.0339 (19)	0.0219 (17)	0.0078 (15)	0.0076 (14)	0.0035 (14)

### Geometric parameters (Å, °)

**Table:**

P1—C1	1.786 (3)	C12—H12	0.9500
P1—C7	1.785 (3)	C13—C18	1.396 (4)
P1—C13	1.794 (3)	C13—C14	1.395 (4)
P1—C19	1.808 (3)	C14—C15	1.392 (5)
S1—C27	1.675 (3)	C14—H14	0.9500
F1—C31	1.370 (4)	C15—C16	1.355 (5)
O1—C23	1.356 (3)	C15—H15	0.9500
O1—H1	0.837 (10)	C16—C17	1.378 (5)
N1—C26	1.281 (4)	C16—H16	0.9500
N1—N2	1.379 (3)	C17—C18	1.395 (4)
N2—C27	1.351 (4)	C17—H17	0.9500
N2—H2	0.881 (10)	C18—H18	0.9500
N3—C27	1.350 (4)	C19—C20	1.513 (4)
N3—C28	1.427 (4)	C19—H19A	0.9900
N3—H3	0.874 (10)	C19—H19B	0.9900
C1—C2	1.390 (4)	C20—C25	1.385 (4)
C1—C6	1.392 (4)	C20—C21	1.400 (4)
C2—C3	1.391 (5)	C21—C22	1.383 (4)
C2—H2A	0.9500	C21—H21	0.9500
C3—C4	1.397 (5)	C22—C23	1.388 (4)
C3—H3A	0.9500	C22—H22	0.9500
C4—C5	1.379 (5)	C23—C24	1.407 (4)
C4—H4	0.9500	C24—C25	1.399 (4)
C5—C6	1.383 (4)	C24—C26	1.453 (4)
C5—H5	0.9500	C25—H25	0.9500
C6—H6	0.9500	C26—H26	0.9500
C7—C12	1.373 (4)	C28—C33	1.374 (5)
C7—C8	1.394 (5)	C28—C29	1.377 (4)
C8—C9	1.365 (5)	C29—C30	1.394 (5)
C8—H8	0.9500	C29—H29	0.9500
C9—C10	1.383 (5)	C30—C31	1.358 (5)
C9—H9	0.9500	C30—H30	0.9500
C10—C11	1.373 (5)	C31—C32	1.372 (5)
C10—H10	0.9500	C32—C33	1.389 (4)
C11—C12	1.390 (4)	C32—H32	0.9500
C11—H11	0.9500	C33—H33	0.9500
			
C1—P1—C7	107.60 (14)	C14—C15—H15	119.7
C1—P1—C13	112.88 (14)	C15—C16—C17	120.9 (3)
C7—P1—C13	109.30 (15)	C15—C16—H16	119.6
C1—P1—C19	108.09 (14)	C17—C16—H16	119.6
C7—P1—C19	110.30 (14)	C16—C17—C18	120.0 (3)
C13—P1—C19	108.65 (14)	C16—C17—H17	120.0
C23—O1—H1	111 (3)	C18—C17—H17	120.0
C26—N1—N2	115.7 (2)	C13—C18—C17	119.4 (3)
C27—N2—N1	119.5 (2)	C13—C18—H18	120.3
C27—N2—H2	119 (3)	C17—C18—H18	120.3
N1—N2—H2	122 (3)	C20—C19—P1	110.05 (19)
C27—N3—C28	127.2 (3)	C20—C19—H19A	109.7
C27—N3—H3	116 (2)	P1—C19—H19A	109.7
C28—N3—H3	116 (2)	C20—C19—H19B	109.7
C2—C1—C6	120.3 (3)	P1—C19—H19B	109.7
C2—C1—P1	123.0 (2)	H19A—C19—H19B	108.2
C6—C1—P1	116.7 (2)	C25—C20—C21	118.8 (3)
C3—C2—C1	119.2 (3)	C25—C20—C19	121.8 (3)
C3—C2—H2A	120.4	C21—C20—C19	119.3 (3)
C1—C2—H2A	120.4	C22—C21—C20	120.7 (3)
C2—C3—C4	120.0 (3)	C22—C21—H21	119.6
C2—C3—H3A	120.0	C20—C21—H21	119.6
C4—C3—H3A	120.0	C21—C22—C23	120.3 (3)
C5—C4—C3	120.5 (3)	C21—C22—H22	119.8
C5—C4—H4	119.7	C23—C22—H22	119.8
C3—C4—H4	119.7	O1—C23—C22	122.6 (3)
C4—C5—C6	119.5 (3)	O1—C23—C24	117.8 (3)
C4—C5—H5	120.2	C22—C23—C24	119.6 (3)
C6—C5—H5	120.2	C25—C24—C23	119.1 (3)
C5—C6—C1	120.4 (3)	C25—C24—C26	121.2 (3)
C5—C6—H6	119.8	C23—C24—C26	119.6 (3)
C1—C6—H6	119.8	C20—C25—C24	121.2 (3)
C12—C7—C8	119.1 (3)	C20—C25—H25	119.4
C12—C7—P1	122.2 (2)	C24—C25—H25	119.4
C8—C7—P1	118.6 (2)	N1—C26—C24	120.7 (3)
C9—C8—C7	120.6 (3)	N1—C26—H26	119.7
C9—C8—H8	119.7	C24—C26—H26	119.7
C7—C8—H8	119.7	N3—C27—N2	113.9 (3)
C8—C9—C10	120.0 (4)	N3—C27—S1	125.7 (2)
C8—C9—H9	120.0	N2—C27—S1	120.3 (2)
C10—C9—H9	120.0	C33—C28—C29	120.2 (3)
C11—C10—C9	120.2 (3)	C33—C28—N3	120.7 (3)
C11—C10—H10	119.9	C29—C28—N3	119.0 (3)
C9—C10—H10	119.9	C28—C29—C30	120.0 (3)
C10—C11—C12	119.6 (3)	C28—C29—H29	120.0
C10—C11—H11	120.2	C30—C29—H29	120.0
C12—C11—H11	120.2	C31—C30—C29	117.9 (3)
C7—C12—C11	120.4 (3)	C31—C30—H30	121.0
C7—C12—H12	119.8	C29—C30—H30	121.0
C11—C12—H12	119.8	C30—C31—F1	118.7 (3)
C18—C13—C14	119.6 (3)	C30—C31—C32	123.8 (3)
C18—C13—P1	120.8 (2)	F1—C31—C32	117.5 (3)
C14—C13—P1	118.7 (2)	C31—C32—C33	117.2 (3)
C15—C14—C13	119.5 (3)	C31—C32—H32	121.4
C15—C14—H14	120.2	C33—C32—H32	121.4
C13—C14—H14	120.2	C28—C33—C32	120.8 (3)
C16—C15—C14	120.6 (3)	C28—C33—H33	119.6
C16—C15—H15	119.7	C32—C33—H33	119.6
			
C26—N1—N2—C27	−172.7 (3)	C14—C13—C18—C17	0.6 (5)
C7—P1—C1—C2	−114.6 (3)	P1—C13—C18—C17	169.7 (3)
C13—P1—C1—C2	6.1 (3)	C16—C17—C18—C13	−0.5 (5)
C19—P1—C1—C2	126.3 (3)	C1—P1—C19—C20	−71.1 (2)
C7—P1—C1—C6	64.5 (3)	C7—P1—C19—C20	171.5 (2)
C13—P1—C1—C6	−174.8 (2)	C13—P1—C19—C20	51.7 (2)
C19—P1—C1—C6	−54.6 (3)	P1—C19—C20—C25	75.2 (3)
C6—C1—C2—C3	3.2 (5)	P1—C19—C20—C21	−100.9 (3)
P1—C1—C2—C3	−177.8 (3)	C25—C20—C21—C22	−2.9 (4)
C1—C2—C3—C4	−0.9 (5)	C19—C20—C21—C22	173.3 (3)
C2—C3—C4—C5	−2.0 (6)	C20—C21—C22—C23	−1.1 (5)
C3—C4—C5—C6	2.6 (5)	C21—C22—C23—O1	−175.4 (3)
C4—C5—C6—C1	−0.3 (5)	C21—C22—C23—C24	4.4 (4)
C2—C1—C6—C5	−2.6 (5)	O1—C23—C24—C25	176.3 (3)
P1—C1—C6—C5	178.2 (2)	C22—C23—C24—C25	−3.5 (4)
C1—P1—C7—C12	−123.2 (3)	O1—C23—C24—C26	−2.2 (4)
C13—P1—C7—C12	113.9 (3)	C22—C23—C24—C26	178.0 (3)
C19—P1—C7—C12	−5.5 (3)	C21—C20—C25—C24	3.8 (4)
C1—P1—C7—C8	59.7 (3)	C19—C20—C25—C24	−172.4 (3)
C13—P1—C7—C8	−63.2 (3)	C23—C24—C25—C20	−0.6 (4)
C19—P1—C7—C8	177.4 (3)	C26—C24—C25—C20	177.9 (3)
C12—C7—C8—C9	0.9 (6)	N2—N1—C26—C24	−177.1 (3)
P1—C7—C8—C9	178.1 (4)	C25—C24—C26—N1	13.1 (4)
C7—C8—C9—C10	1.2 (7)	C23—C24—C26—N1	−168.4 (3)
C8—C9—C10—C11	−1.6 (7)	C28—N3—C27—N2	170.9 (3)
C9—C10—C11—C12	−0.1 (6)	C28—N3—C27—S1	−9.9 (5)
C8—C7—C12—C11	−2.6 (6)	N1—N2—C27—N3	3.7 (4)
P1—C7—C12—C11	−179.7 (3)	N1—N2—C27—S1	−175.5 (2)
C10—C11—C12—C7	2.2 (6)	C27—N3—C28—C33	−47.5 (5)
C1—P1—C13—C18	51.5 (3)	C27—N3—C28—C29	137.0 (3)
C7—P1—C13—C18	171.2 (3)	C33—C28—C29—C30	2.2 (5)
C19—P1—C13—C18	−68.4 (3)	N3—C28—C29—C30	177.7 (3)
C1—P1—C13—C14	−139.3 (3)	C28—C29—C30—C31	0.7 (5)
C7—P1—C13—C14	−19.6 (3)	C29—C30—C31—F1	177.3 (3)
C19—P1—C13—C14	100.8 (3)	C29—C30—C31—C32	−2.8 (6)
C18—C13—C14—C15	0.0 (5)	C30—C31—C32—C33	1.8 (5)
P1—C13—C14—C15	−169.3 (3)	F1—C31—C32—C33	−178.3 (3)
C13—C14—C15—C16	−0.8 (5)	C29—C28—C33—C32	−3.2 (5)
C14—C15—C16—C17	0.8 (6)	N3—C28—C33—C32	−178.6 (3)
C15—C16—C17—C18	−0.2 (6)	C31—C32—C33—C28	1.2 (5)

### Hydrogen-bond geometry (Å, °)

**Table:**

*D*—H···*A*	*D*—H	H···*A*	*D*···*A*	*D*—H···*A*
N3—H3···N1	0.87 (1)	2.16 (3)	2.580 (4)	109 (3)
O1—H1···Cl1	0.84 (1)	2.17 (1)	3.005 (2)	173 (4)
N2—H2···S1^i^	0.88 (1)	2.58 (2)	3.429 (3)	162 (3)
C6—H6···Cl1^ii^	0.95	2.69	3.572 (3)	154
C19—H19a···Cl1^ii^	0.99	2.51	3.488 (3)	168
C19—H19b···Cl1^iii^	0.99	2.59	3.553 (3)	165

Symmetry codes: (i) −*x*+1, −*y*+1, −*z*+1; (ii) *x*, −*y*+3/2, *z*−1/2; (iii) −*x*+2, *y*+1/2, −*z*+3/2.
